# Different effects of flash-grab and frame stimuli on position shift and shape distortion

**DOI:** 10.1167/jov.26.3.1

**Published:** 2026-03-02

**Authors:** Mohammad Shams, Aurore Maloh, Peter J. Kohler, Patrick Cavanagh

**Affiliations:** 1 Department of Psychology, Glendon College, York University, Toronto, Ontario, Canada; 2 Centre for Vision Research, York University, Toronto, Ontario, Canada; 3 Department of Psychology, York University, Toronto, Ontario, Canada

**Keywords:** motion perception, perceived position, motion-induced position shift, motion-induced shape distortion

## Abstract

In the flash-grab effect, an object flashed on a moving background appears to be shifted in the direction of the motion. The same background motion also distorts the flashed object's perceived shape. An even greater shift in the perceived location is produced by the frame effect, raising the question of whether it also produces a shape distortion. This phenomenon is important because the frame effect has been linked to perceptual stabilization during eye movements where the whole visual field acts as the frame. We found that, unlike the flash-grab case, shape was preserved for the frame effect to a much greater extent than for the flash-grab. Next, we tested the extent to which shape distortions could be predicted from the size of the shifts in position of individual shape elements. We found that observed distortions were weaker than predicted distortions for the frame effect, but stronger for the flash-grab stimulus. Finally, we examined whether the greater shape distortion for the flash-grab was due to the nature of the background motion (rotation vs. translation) or the aperture within which the background motion was presented (circular vs. rectangular). We found that both factors contributed to greater shape distortion. Our findings show that motion-induced shape distortions are not solely based on the individual position shifts of the shape elements when tested in isolation. The shape preservation for the frame effect may be achieved through engaging shape-based mechanisms tuned to the dynamics of saccadic eye movements.

## Introduction

Motion can dramatically alter the perceived location of briefly presented objects, a phenomenon known as the motion-induced position shift, for example, where a static probe dot flashed near a moving object is perceived as displaced in the direction of motion (Whitney & Cavanagh, 2000). Two stimuli are particularly strong at demonstrating such effect. In the first—the flash-grab effect (Cavanagh & Anstis, 2013)—a background pattern rotates back and forth, and a probe dot is flashed at the moment the background reverses direction. The probe appears to be shifted in the direction of the motion after the reversal (Supplementary Movie S1). In the second, the frame effect (Cavanagh et al., 2022; Duncker, 1929; Özkan, Anstis, ’t Hart, Wexler, & Cavanagh, 2021; Wong & Mack, 1981), a frame moves back and forth, and two probe dots are flashed at the same location, one at each motion reversal, one at the left edge of the frame and the other at the right edge. Despite being aligned on the screen, the probes appear to be separated by nearly the full distance of the frame's motion (Supplementary Movie S2), as if they are perceived in the frame's coordinates.

These motion-induced shifts have not only been of interest for understanding visual motion processing, but also for their potential relevance to visual stability: During saccadic eye movements, the retinal image undergoes frequent and substantial shifts, yet the visual world is perceived as stable with object positions relative to the visual scene and shapes remarkably undistorted. The frame effect, in particular, has been suggested to play a role in this stabilization process (Özkan et al., 2021).

Recent evidence has shown that the flash-grab stimulus does more than shift perceived position: It also strongly distorts the shape of visual objects (Adamian, Anstis, & Cavanagh, 2023). However, it is unknown whether the frame stimulus similarly alters perceived shape. If a similar distortion were seen for both effects, it would suggest that they share common mechanisms. However, if the frame effect is related to mechanisms that support perceptual stability during eye movements, one might expect that, unlike the flash-grab effect, it should preserve object shape, because little or no distortion of object shape is seen contingent on eye movements (Matsumiya & Uchikawa, 2001).

To test this idea, we conducted three experiments. In Experiment 1 we compared the flash-grab and frame stimuli directly with determine how each affects perceived shape. In Experiment 2, we tested the perceived location of the individual probe dots comprising a shape, presented one at a time, to examine whether the difference in shape distortion could be explained by local position shifts alone. Finally, in Experiment 3, we systematically varied background motion (rotation vs. translation) and the aperture within which the motion was presented (circular vs. rectangular) to determine whether these factors underlie any differential effects of the two stimuli on shape perception.

## Experiment 1

In this experiment, we asked whether the frame stimulus distorts shapes (Supplementary Movie S3) and, if so, whether the distortion is different from that caused by the flash-grab stimulus (Supplementary Movie S4).

### Methods

#### Participants

Sixteen participants were recruited from York University, Toronto, Ontario, Canada. All participants were naïve to the purpose of the study, except for one. One participant was excluded owing to self-reported poor vision experienced during the task. The remaining 15 participants (mean age, 21.5 ± 5.1years) had normal or corrected-to-normal vision and reported no difficulties in perceiving the probes during the post-experimental briefing. All analyses were conducted on this final sample. The study was approved by the Human Participants Review Sub-Committee of York University's Ethics Review Board. Written, informed consent was obtained from each participant prior to their experimental sessions and participants were compensated with credit points. All methods of study were carried out in accordance with the Declaration of Helsinki guidelines and regulations (2003).

#### Stimulus and task

**Figure 1.**
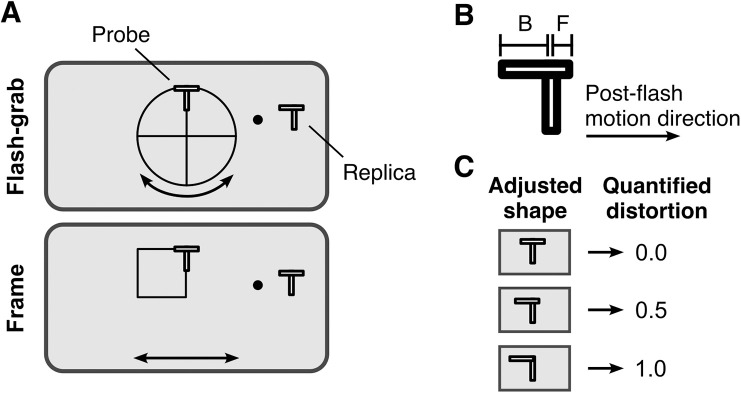
Stimulus design and shape distortion quantification in Experiment 1. (**A**) Flash-grab stimulus and frame stimulus along with the T-shaped probe and the T-shaped replica with an adjustable horizontal component. (**B**) Quantification of the shape distortion. Letters F and B indicate the length of the front and back parts, respectively. (**C**) Three examples of the adjusted replica and their corresponding quantified distortion magnitude. In all three panels, a rightward post-flash motion direction was assumed.

All stimuli were presented on an LCD monitor (21″, 1,920 × 1,080 pixels, 60 Hz) against a gray background (28 cd/m^2^; RGB: 128, 128, 128; eight-bit scale) in a dark room. The experiment included two conditions: flash-grab and frame. In the flash-grab condition, a four-sectored disc (radius, 5 dva) (Figure 1A) appeared at the center of the screen and rotated around its center. The disc initially rotated 90° in a randomly chosen direction over 400 ms, then reversed direction to return to its starting position over the next 400 ms. Each reversal was preceded by a 50-ms pause. In the frame condition, a square (width, 5 dva) moved horizontally over a 5-dva path for 400 ms. The motion started randomly from one of two positions: either with the left edge of the square at the center of the screen, moving leftward until its right edge reached the center, or the reverse.

Starting from the third motion pause, a T-shaped probe appeared above center during every other pause for entire 50 ms of the pause. The T-probe consisted of two bars with a fixed spatial relation to each other: a vertical bar and a horizontal bar of equal size (length, 1.5 dva; width, 0.3 dva), with the horizontal bar centered on top of the vertical bar. The vertical component always appeared aligned with an edge of the moving stimulus, either at the border between two sectors in the flash-grab condition or on one of the vertical edges of the square in the frame condition. Both components of the T-shaped probe had a white surface (89 cd/m^2^) surrounded by a black edge (1 cd/m^2^).

Throughout each trial, participants maintained their fixation on a dot (radius, 0.25 dva) located 6.5 dva to the right and 3 dva above the screen center, with a random vertical offset of ±1 dva added on each trial. A replica of the probe appeared to the right of the fixation dot. Because we were interested in the relative shift between the horizontal and vertical components of the T-probe, we kept the vertical component fixed (at a 2-dva distance from the fixation dot) while the participants used a mouse to adjust the horizontal position of the horizontal component in the replica. Once the replica matched their perceived shape of the probe, they confirmed their response with a key press. To prevent any decision bias, the starting position of the horizontal component at each trial was randomly offset by ±0.75 dva relative to the vertical component.

Each participant performed a total of 40 trials, pseudo-randomly distributed across four conditions: two stimulus types (flash-grab, frame) and two post-flash motion directions (left, right).

Here and in the following experiments, the data violated the normality assumption required by parametric tests (Kolmogorov–Smirnov test: flash-grab, *p* < 0.001; frame, *p* < 0.001), therefore, we performed Wilcoxon signed rank tests to compare the distortions induced by the flash-grab and frame.

### Results

The perceived shape distortion (*D*) was quantified as:

$$D=\frac{F-B}{F+B},$$

where *F* and *B* represent the horizontal distances from the vertical bar to the front and back corners of the horizontal bar in the adjusted replica, respectively (see Figures 1B and 1C). Positive values indicate a distortion in the direction of post-flash motion.

In the flash-grab stimulus, we observed a difference in the magnitude of the induced shape distortion between the centrifugal (median, 0.45) and centripetal (median, 0.30) post-flash motion direction (Wilcoxon signed-rank test: *W* = 12; *p* < 0.004; *r* = 0.8), but we did not find any evidence for a similar difference in the frame stimulus (Wilcoxon signed-rank test: *W* = 45; *p* < 0.421; *r* = 0.25). For further analyses, the responses were pooled across the two post-flash directions after correcting for direction and the mean distortion was computed for each participant.

**Figure 2.**
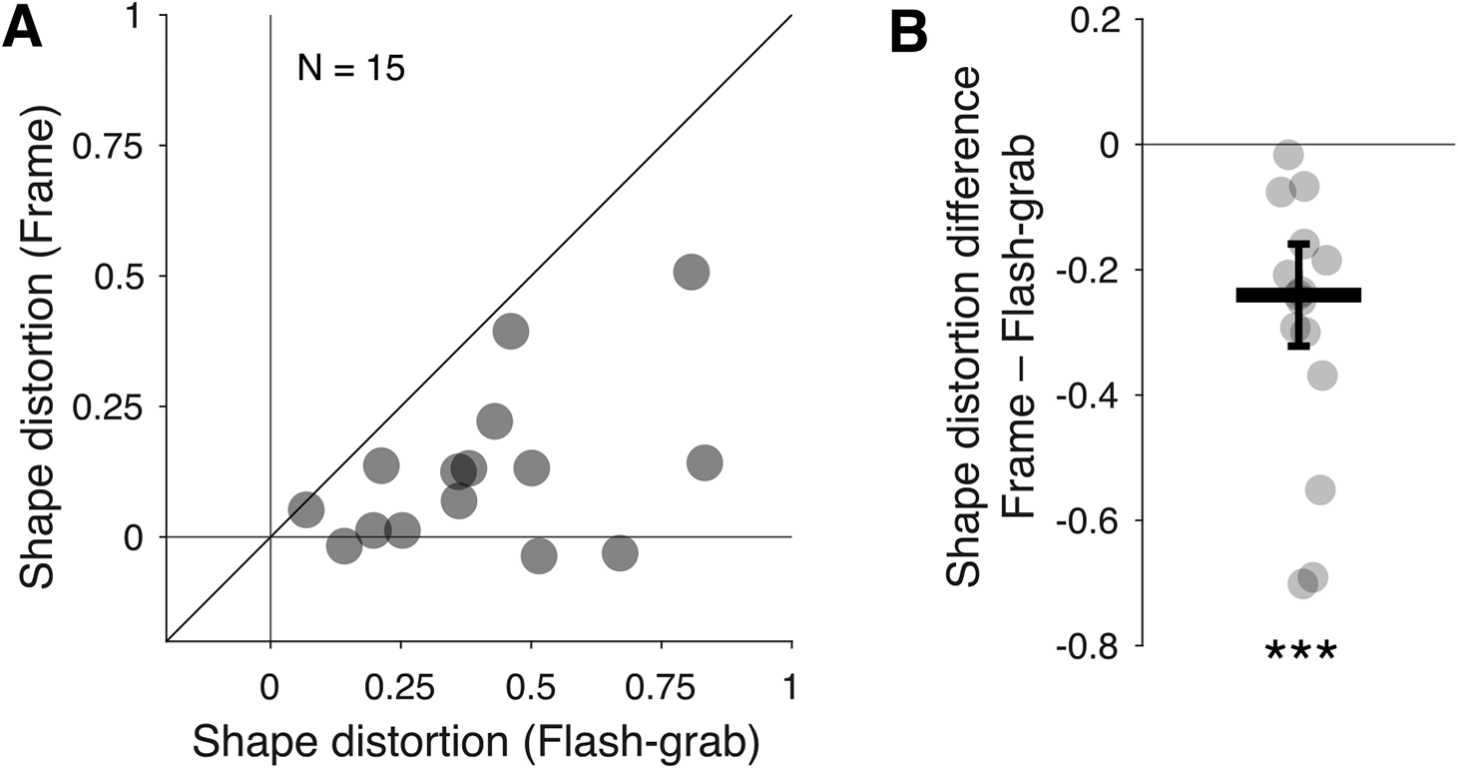
Perceived shape distortion magnitudes in the frame and flash-grab stimuli. (**A**) Each point represents one participant's distortion in the flash-grab (*x*-axis) and frame (*y*-axis) condition. The oblique line indicates the identity line. (**B**) The difference in the induced shape distortion is shown for each participant. Negative values indicate smaller distortion in frame condition. The thick horizontal line indicates the median and the error bar ± median absolute difference. ***p ≤ 0.001.

The frame stimulus induced a shape distortion with a median of 0.12 and the flash-grab stimulus a distortion with a median of 0.38 (Figure 2A). The shape distortion induced by the frame stimulus was smaller than that induced by the flash-grab stimulus across all participants, with a median difference of 0.24 (Figure 2B; Wilcoxon signed-rank test: *W* = 0; *p* < 0.001; *r* = 1).

These medians were averaged across the two directions of the background motion following the presentation of the probe. However, for the flash-grab results, there was a significant difference in the magnitude of the induced shape distortion when the background moved rightward, toward fixation after the probe's presentation compared with when it moved leftward, away from fixation (median_right_, 0.3; median_left_, 0.45; Wilcoxon signed-rank test, *W* = 12; *p* < 0.004; *r* = 0.8). This outcome is similar to the asymmetry reported for the flash lag effect (Shi & Nijhawan, 2008). In contrast, we found no significant effect for the post-flash motion direction for the frame stimulus (median_right_, 0.03; median_left_, 0.1; Wilcoxon signed-rank test: *W* = 45; *p* < 0.421; *r* = 0.25). The results from the two post-flash directions have been pooled in the following analyses.

In this experiment, as in Adamian et al. (2023), we found that the flash-grab stimulus can distort perceived shape. Those authors proposed that the distortion was a consequence of local position shifts that were larger for the parts of the shape closest to salient edges (e.g., the border between two sectors). In contrast, the frame stimulus led to significantly weaker shape distortions. This dissociation suggests that the frame effect may preserve the spatial configuration of shape elements and that it operates to some extent through different mechanisms than those underlying the flash-grab effect.

## Experiment 2

To test whether the shape distortions arose from differences in local shifts across the shape (Adamian et al., 2023), single probe dots were presented (one at a time) that matched the center and end points of the top horizontal segment of the T-shaped object used in Experiment 1 (Supplementary Movies S5 and S6). This design allowed us to measure motion-induced position shifts at the locations of the T-shape's individual parts under both the flash-grab and frame conditions. By comparing these locally sampled positions with the distortion observed when the full shape was presented simultaneously (as in Experiment 1), we can assess whether the perceived distortion of the whole shape could be explained by the aggregated position shifts of its parts. This approach also provided a more direct measure of the magnitude of position shifts induced by the two stimuli, building on recent findings suggesting that single-element probes can underestimate the full extent of frame-induced position shift (Shams, Kohler, & Cavanagh, 2024).

### Methods

The participants in Experiment 2 were the same individuals who completed Experiment 1.

#### Stimulus and task

**Figure 3.**
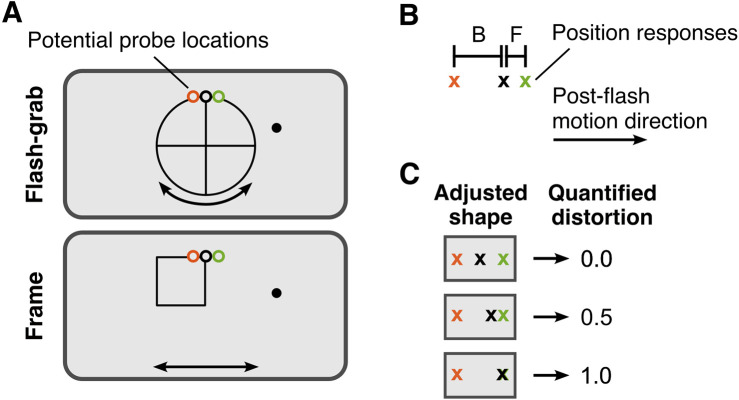
Stimulus design and shape distortion quantification in Experiment 2. (**A**) Flash-grab stimulus and frame stimulus along with the three potential probe locations. The three probe colors orange, black, and green represent backward, central, and frontal probe locations. Only one probe was shown on each trial. (**B**) Calculation of the predicted shape distortion based on the position response locations. Letters F and B indicate the frontal and backward position response locations to the central position response location, respectively. (**C**) Three examples of the position responses and their corresponding quantified distortion magnitude. In all three panels, a rightward post-flash motion direction was assumed.

The two moving backgrounds for the flash-grab and the frame conditions, as well as their kinematics, were identical to those of Experiment 1. In every trial, one probe dot (radius, 0.25 dva) appeared for 50 ms at one of the three potential locations corresponding with the two tails and the center of the horizontal component of the T-shaped probe in Experiment 1 (Figure 3A). The stimulus oscillated four times and the probe flashed at the beginning of each cycle, except for the first one. Probes flashed at 5 dva above the center of the screen with a horizontal offset of −0.75, 0, or 0.75 dva from the midline. After that, the screen went blank the mouse cursor appeared, and participants reported the location of the flashed probe by a mouse click.

Each participant performed a total of 120 trials, pseudo-randomly distributed across 12 conditions: 2 stimulus types (flash-grab, frame), 2 post-flash motion directions (left, right), and 3 probe locations.

### Results

**Figure 4.**
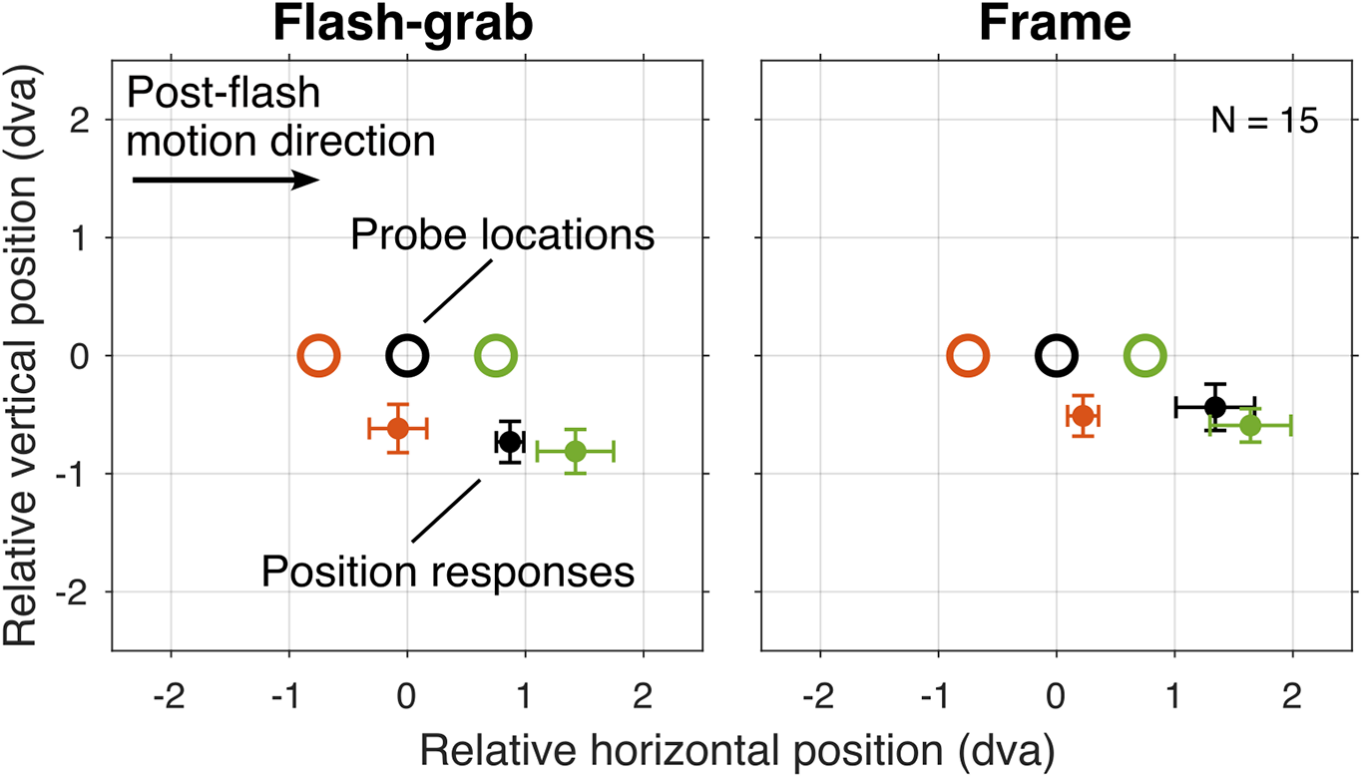
Stimulus design and position responses in Experiment 2. Open circles represent the flashed probes. Dots with error bars represent the median position responses ± median absolute difference across participants corresponding to each probe location of the same color. In each trial, the probe was presented as a red dot; the three colors used here are only for visual purposes.

The locations indicated by the mouse clicks were pooled across the two post-flash directions after correcting for directionality, and the mean reported location corresponding with each of the three probe locations was computed for each participant (Figure 4). The average position shift induced by the frame stimulus was larger than that induced by the flash-grab stimulus.

**Figure 5.**
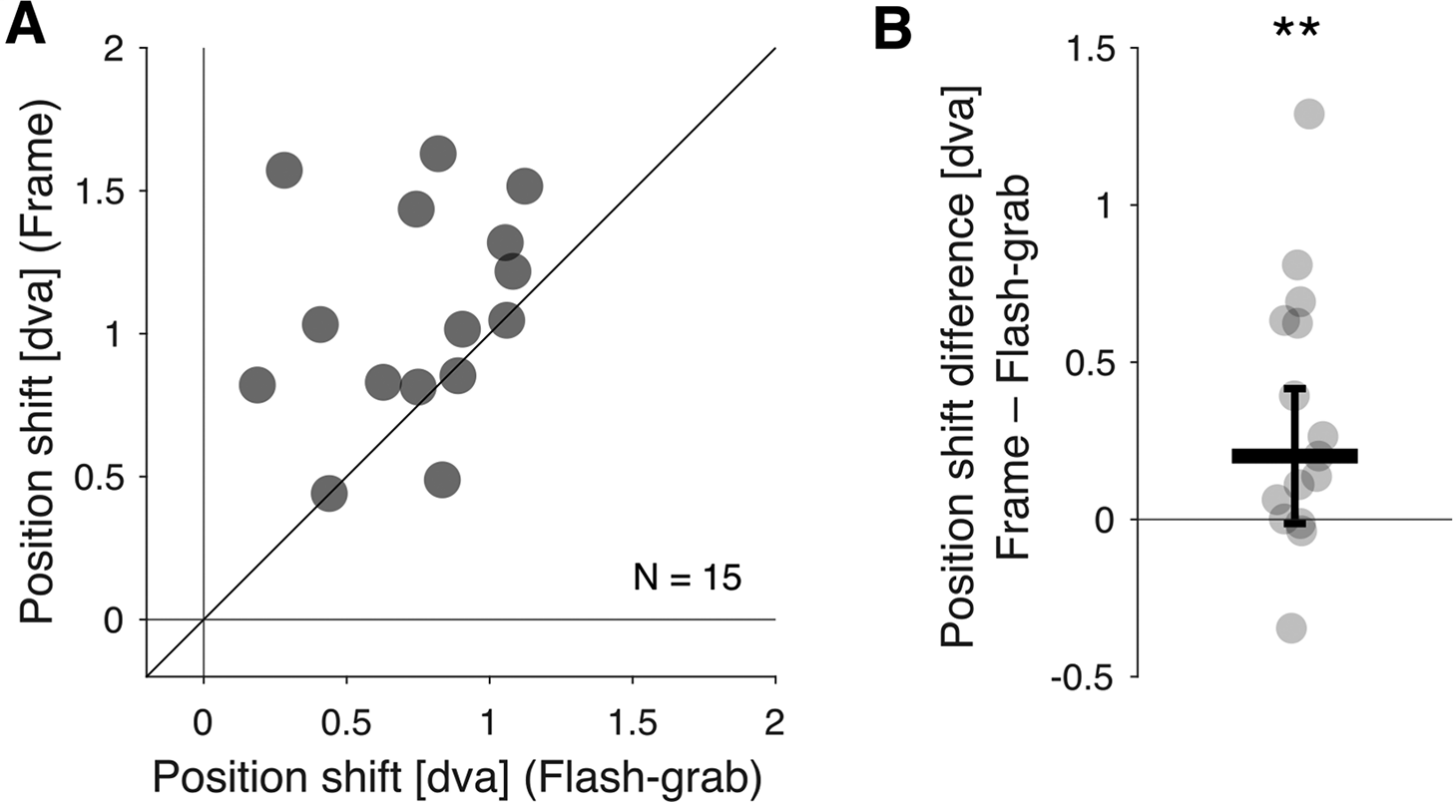
Perceived position shift of individual probe dots in the frame and flash-grab stimuli. (**A**) Each point represents one participant's average position response errors for all probes in the flash-grab (*x*-axis) and frame (*y*-axis) condition. The oblique line indicates the identity line. (**B**) The difference in induced position shift is shown for each participant. Positive values indicate greater distortion in frame condition. The thick horizontal line indicates the median and the error bar ± median absolute difference. **p ≤ 0.01.

The median position shift was 1.03 dva with the frame stimulus and 0.82 dva with the flash-grab stimulus (Figure 5A) and the flash-grab median difference of 0.21 dva was significant (Figure 5B; Wilcoxon signed-rank test: *W* = 14; *p* = 0.007; *r* = 0.77).

Based on the average reported locations corresponding with the three probes, we calculated the predicted distortion using the method described in Experiment 1, where the distance between the average position responses corresponding with the back probe and the front probe to that of the center probe constituted *B* and *F* (Figures 3B and 3C).

**Figure 6.**
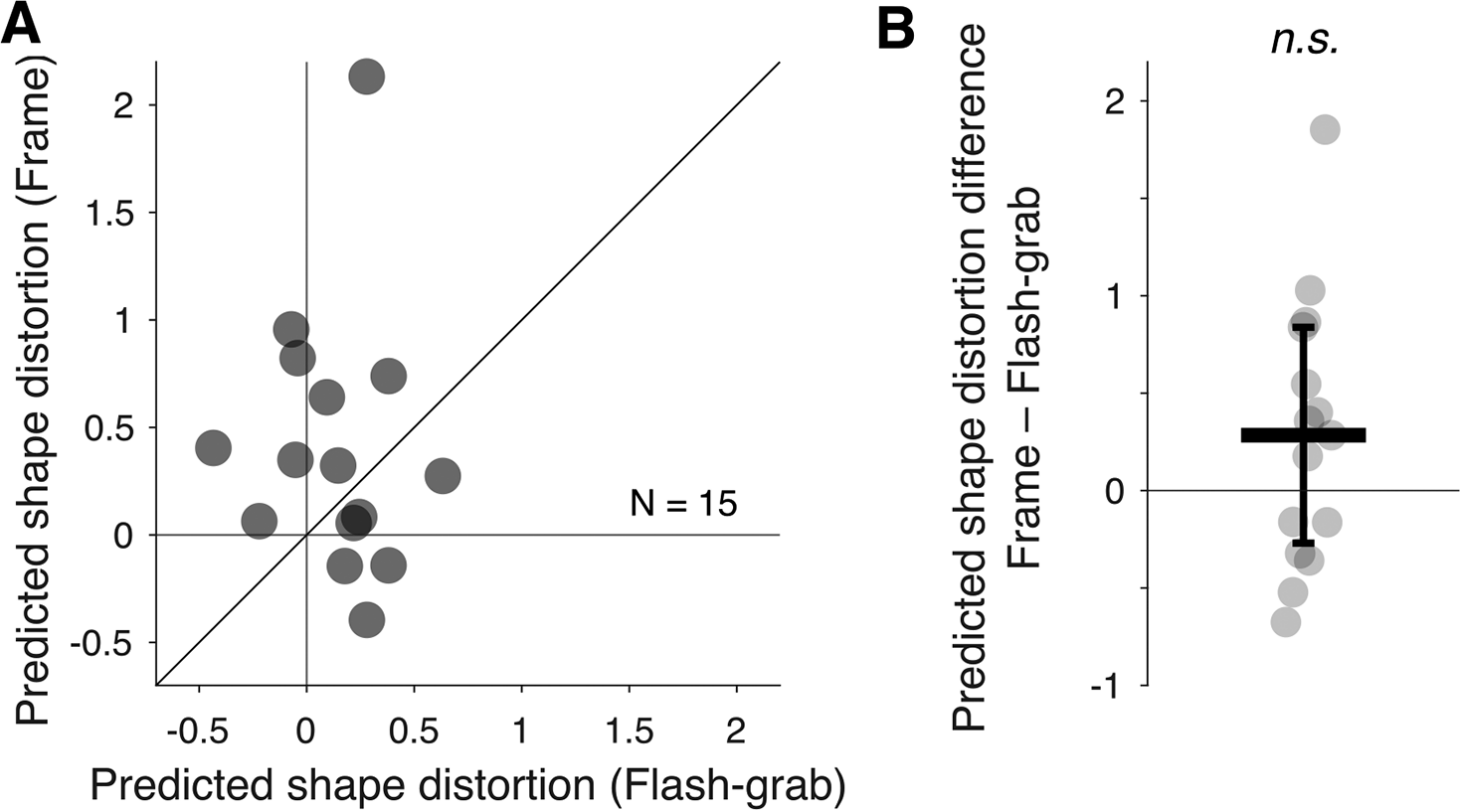
Predicted shape distortion based on the perceived position shift of individual probe dots in the frame and flash-grab stimuli. (**A**) Each point represents one participant's predicted shape distortion in the flash-grab (*x*-axis) and frame (*y*-axis) condition. The oblique line indicates the identity line. (**B**) The difference in predicted shape distortion is shown for each participant. Positive values indicate greater distortion in frame condition. The thick horizontal line indicates the median and the error bar ± median absolute difference. *n*.*s*., not significant.

The predicted shape distortion induced by the frame stimulus had a median of 0.32 and that induced by the flash-grab stimulus had a median of 0.18 (Figure 6A). We did not find a significant difference in the predicted shape distortion between the frame and flash-grab stimuli (Figure 6B) (median difference, 0.28; Wilcoxon signed-rank test: *W* = 35; *p* = 0.169; *r* = 0.42).

**Figure 7.**
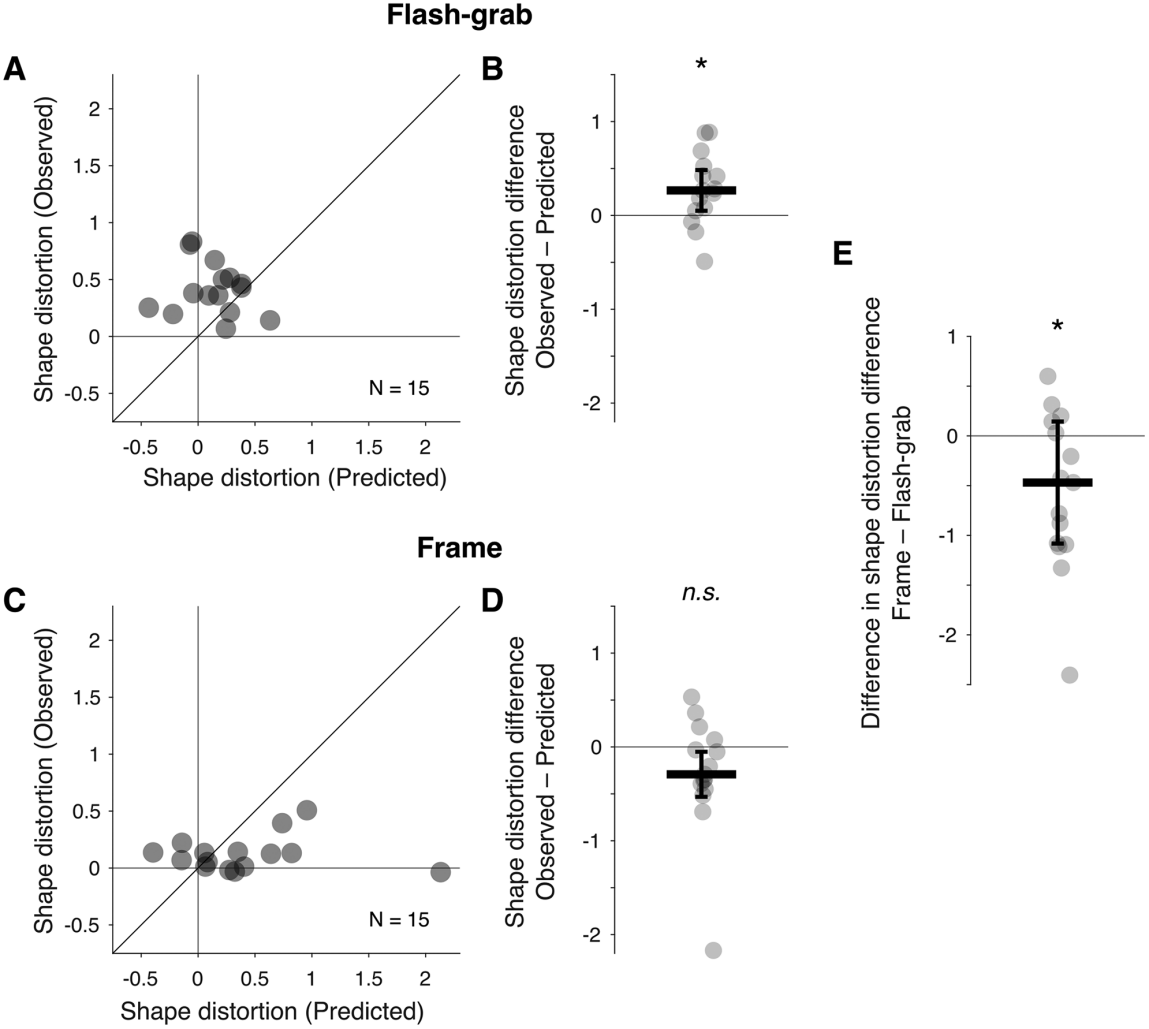
Observed and predicted shape distortions in the flash-grab and frame conditions. (**A**) Each point represents one participant's predicted (*x*-axis) and observed (*y*-axis) shape distortion in the flash-grab condition. The oblique line indicates the identity line. (**B**) Difference between observed and predicted distortion in the flash-grab condition, with positive values indicating stronger distortion when directly observed. (**C** and **D**) Same as (**A** and **B**), but for the frame condition. (**E**) Difference of the distortion differences between conditions, computed as (observed − predicted) in the frame condition minus (observed − predicted) in the flash-grab condition. The thick horizontal lines indicate the median and the error bars ± median absolute difference. *p ≤ 0.05. *n*.*s*., not significant.

We found no meaningful correlation between the observed distortions from Experiment 1 and distortions predicted from individual points here, neither in flash-grab condition (Figure 7A) (Spearman's ρ = −0.17; *p* = 0.54), nor in frame (Figure 7C) (Spearman's ρ = −0.04; *p* = 0.88). The observed shape distortion induced by the flash-grab stimulus measured in Experiment 1 was larger than the shape distortion predicted from the individual points here (Figure 7B) (median difference, 0.27; Wilcoxon signed-rank test: *W* = 17; *p* = 0.012; *r* = 0.72). In contrast, the observed shape distortion induced by the frame stimulus from Experiment 1 tended to be smaller than shape distortion predicted from the individual points here (Figure 7D) (median difference, − 0.29; Wilcoxon signed-rank test: *W* = 30; *p* = 0.095; *r* = 0.5). The two stimuli differed significantly regarding the difference between their induced observed and predicted shape distortion. The median difference between the observed (Experiment 1) and predicted distortions (Experiment 2) in the frame stimulus was 0.47 smaller than the corresponding difference for the flash-grab stimulus (Figure 7E) (Wilcoxon signed-rank test: *W* = 19; *p* = 0.018; *r* = 0.68).

The results of Experiment 2 show that the flash-grab stimulus is more effective at distorting perceived shape than at shifting the perceived position of individual elements of the shape. In contrast, the frame stimulus, despite producing stronger position shifts than the flash-grab stimulus, was better at preserving the perceived shape of the object. Moreover, the flash-grab stimulus produced greater distortion when the entire shape was visible than when it was predicted from individual probe positions, whereas the frame stimulus preserved the perception of a complete shape better than the shape predicted from the same probes.

## Experiment 3

The differences in the position shifts and shape distortion between the flash-grab stimulus and the frame stimulus seen in Experiments 1 and 2 suggest that the two effects are, to some extent, driven by distinct mechanisms. These two stimuli differ along two key factors: motion trajectory (rotation vs. translation) and the aperture within which the motion is presented (circular vs. rectangular). However, because both factors varied simultaneously, it is not clear which of them (or their combination) was responsible for the observed difference in distortion. To isolate the contributions of motion type and aperture, in Experiment 3, we varied these two factors, creating four distinct stimuli that combined rotation or translation motion with circular or rectangular shape and added the standard frame stimulus as the reference stimulus for comparison.

### Methods

The participants in Experiment 3 were the same individuals who completed Experiments 1 and 2.

#### Stimulus and task

**Figure 8.**
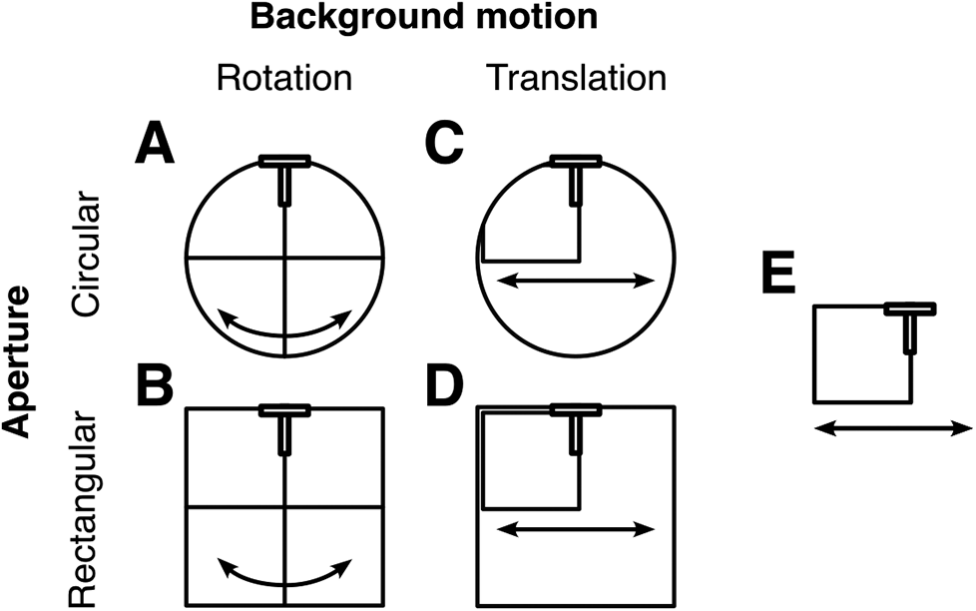
Stimuli used to test the influence of background motion trajectory and aperture shape on perceived shape distortion in Experiment 3. Five stimulus configurations were used: (**A**) rotating cross within circular aperture (flash-grab stimulus), (**B**) rotating cross within rectangular aperture, (**C**) translating square within circular aperture, (**D**) translating frame within rectangular aperture, and (**E**) translating frame (frame stimulus).

The stimulus and task design of this experiment were an extension of Experiment 1. We created a 2 × 2 design by varying background motion and aperture shape: a rotating cross inside a circular aperture (Figure 8A; Supplementary Movie S4), a rotating cross inside a rectangular aperture (Figure 8B; Supplementary Movie S7), a translating square inside a circular aperture (Figure 8C; Supplementary Movie S8), and a translating square inside a rectangular aperture (Figure 8D; Supplementary Movie S9). To complete the picture and allow further comparisons, we included the frame stimulus (Figure 8E; Supplementary Movie S3) as it was used in the previous two experiments. The task procedure was the same as the one used in Experiment 1.

Each participant performed a total of 100 trials, pseudo-randomly distributed across 10 conditions: 5 stimulus types and 2 post-flash motion directions (left and right).

### Results

**Figure 9.**
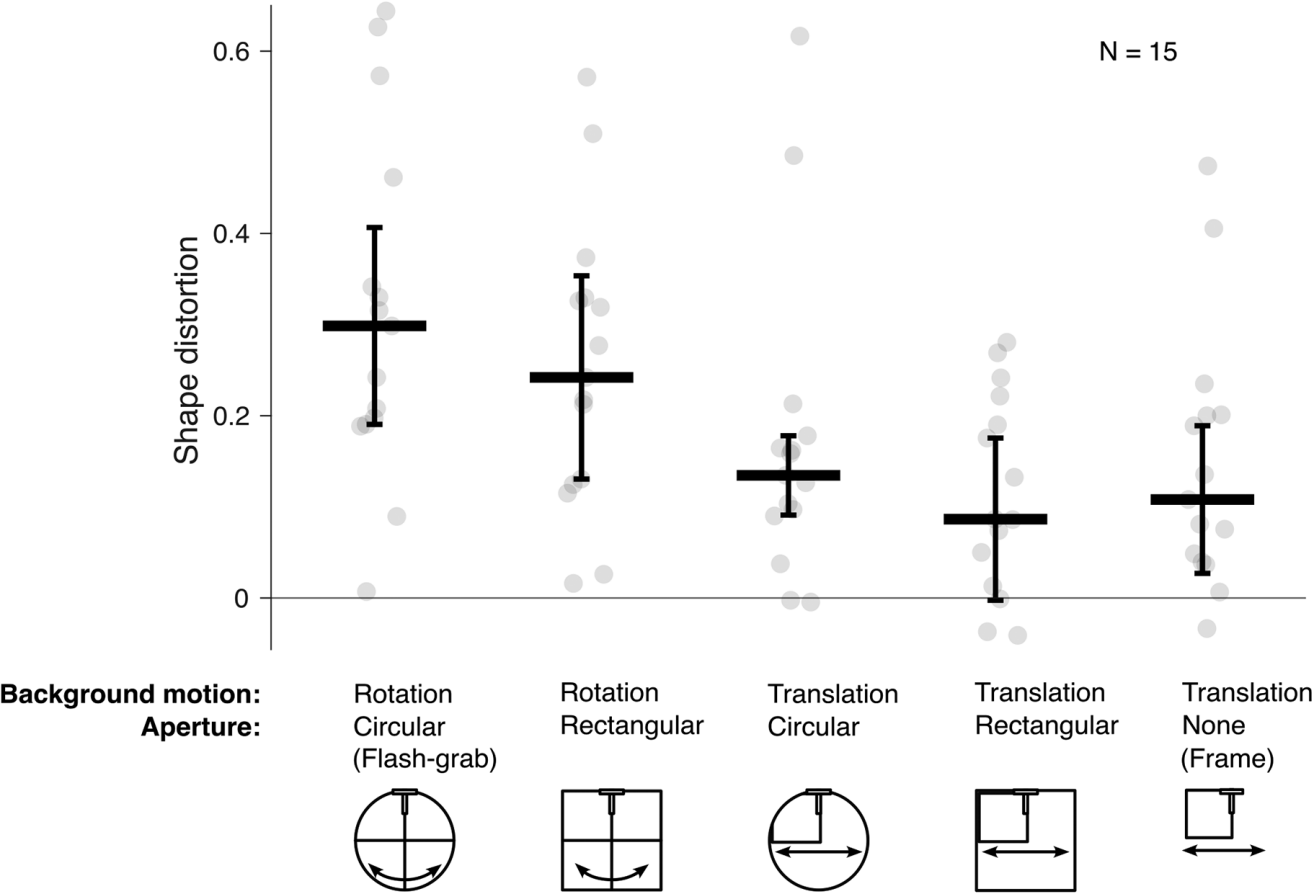
Shape distortion across five background conditions. Shape distortion values are plotted for each of the five stimulus types, with each point representing the mean response of a single participant. Thin lines connect responses from the same participant across conditions. The thick horizontal lines indicate the median and the error bars ± median absolute difference.

Shape distortion varied systematically across the five stimuli (Figure 9). A Friedman test revealed a significant effect of stimulus condition on perceived distortion, χ^2^(4) = 27, *p* < 0.001, confirming that the five conditions differed in their ability to induce shape distortion. The greatest distortion was induced by the flash-grab stimulus (median, 0.30), where a rotating cross appeared inside a circular aperture and the smallest distortion by the translating square inside a rectangular aperture (median, 0.09).

From Figure 9, it is noticeable that stimuli involving rotational motion generally produced stronger distortions than those involving translation, and circular apertures tended to elicit greater distortions than rectangular ones. To test the individual contributions of background motion and background aperture, responses were collapsed across apertures and motion types, respectively, excluding the control frame condition. Shape distortion was significantly greater for stimuli involving rotation compared with translation stimulus, with a median difference of 0.13 (Wilcoxon signed-rank test: *W* = 2; *p* < 0.001; *r* = 0.97), and for stimuli with circular apertures compared with rectangular apertures with a median difference of 0.05 (Wilcoxon signed-rank test: *W* = 16; *p* = 0.01; *r* = 0.73).

**Figure 10.**
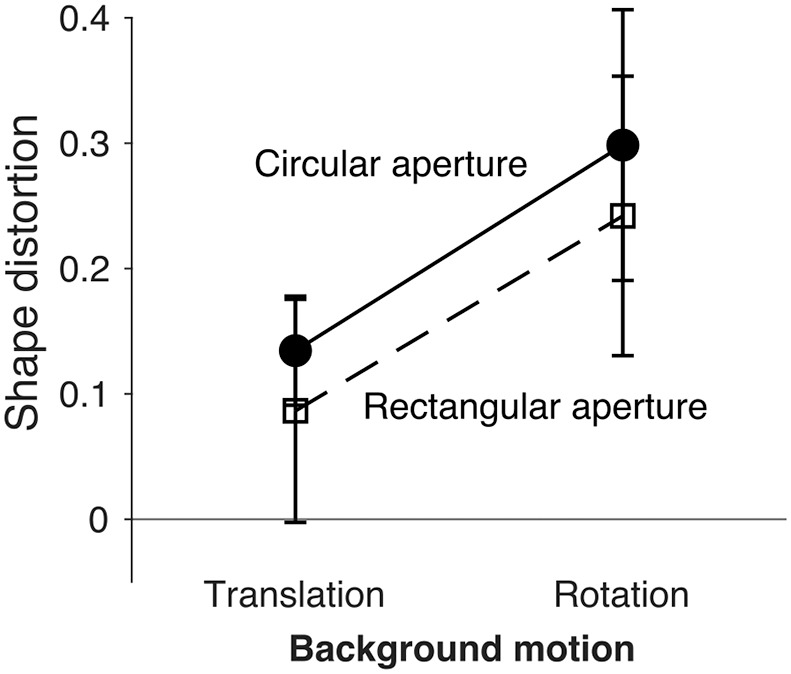
Interaction between background motion (translation vs. rotation) and aperture shape (circular vs. rectangular) on perceived shape distortion. Interaction plot showing median shape distortion for circular (continuous line with filled circles) and rectangular (dashed line with open squares) apertures under translation and rotation conditions. Error bars indicate ± median absolute difference.

We ran a 2 × 2 repeated-measures analysis of variance, with factors of motion type (rotation vs. translation) and motion aperture (circular vs. rectangular) revealed significant main effects of motion, *F*(1, 14) = 27.26, *p* < 0.001, $\eta_{p}^{2}$ = 0.66, and shape, *F*(1, 14) = 6.79, *p* = 0.021, $\eta_{p}^{2}$ = 0.33. The interaction between background motion and aperture shape was not significant (Figure 10), *F*(1, 14) = 0.038, *p* = 0.849, $\eta_{p}^{2}$ = 0.003. These results confirm the outcome of the non-parametric analysis and suggest that both background motion and aperture influence shape distortion and found no evidence that these effects depend on each other.

## Discussion

In this study, we examined how motion-induced position shifts influence the perception of object shape, focusing on two well-established stimuli: the flash-grab stimulus (Cavanagh & Anstis, 2013) and the frame stimulus (Özkan et al., 2021). Consistent with previous findings (Adamian et al., 2023), we found that the flash-grab stimulus induces substantial shape distortion (Figure 2). In contrast, despite producing larger position shifts than the flash-grab stimulus for individual probe dots (Figure 5), the frame stimulus preserved object shape to a significantly greater extent (Figure 2). This result aligns with the hypothesis that the frame effect is supported by perceptual mechanisms involved in visual stabilization across saccades (Özkan et al., 2021). The visual system maintains a coherent and undistorted perception of object shape despite the retinal displacements during saccadic eye movements (Matsumiya & Uchikawa, 2001). It appears that linear motion trajectories (like those of saccades but slower here) are more effective at preserving shape structure than the rotational trajectories of the flash-grab stimulus.

Interestingly, the shape distortion predicted from the localization of individual elements of the shape (Experiment 2) was comparable across the two stimuli (Figure 6). Moreover, we observed no significant correlation between predicted distortion (Experiment 2) and observed distortion (Experiment 1), neither with the flash-grab stimulus (Figure 7A) nor with the frame stimulus (Figure 7C). Adamian et al. (2023) proposed that a gradient of motion-induced local shifts could account for the motion-induced shape distortion. However, our predicted–observed test does not support this account. This apparent discrepancy between the global distortion of a shape and the local position shifts of its elements is reminiscent of Morinaga's paradox of displacement (Morinaga & Ikeda 1965). In that study, observers reported a global distortion of static geometrical shapes, whereas the element-wise presentation of the shapes did not undergo the same spatial shift. These results suggest that the differences in perceived shape distortion observed in Experiment 1 are not simply a result of local mislocalizations induced by motion as had been suggested by Adamian et al. (2023), but likely involve additional mechanisms beyond low-level position shifts.

One difference between the flash-grab and frame stimuli in in Experiments 1 and 2 is the extension of the contour to the left and right of the probe. In Experiment 3, however, we did not find any difference (*p* = 0.252) between the regular frame effect (Figure 9, 5th bin; contour in motion and on one side of probe only) compared with when the frame moved within a rectangular aperture (Figure 9, 4th bin). Moreover, although the proximity of the probe to the moving vertical background contour is indeed an important factor, the proximity to the horizontal contour does not seem to be important. In particular, Adamian et al. (2023) presented the T probe inside a rotating sectored ring, inward of the circular contour, and still found a strong illusion. These observations suggest that difference between the flash-grab and the frame stimuli did not arise from the nature of the top edge (extended or not, moving or not).

The motion-induced distortion effect appears to have quite large variation across individuals and the source of this variation is not clear. Nevertheless, despite the large range of settings, the differential effects of flash-grab and frame stimuli on shape distortion and position shift were highly consistent across individuals and these are the differences on which the conclusions of the article are based. All participants reported a larger distortion in the flash-grab condition than in the frame condition (Experiment 1, Figure 2) and, except for one, all participants reported a larger or equal position shift in the frame condition than the flash-grab condition (Experiment 2, Figure 5). In the third experiment, the range of settings was again large but consistent. When the background motion was rotational, all participants reported a distortion in the same direction. When the background motion was translational, all but one or two participants (depending on the condition) reported a distortion in the same direction.

Why does the frame stimulus preserve shape more effectively than the flash-grab stimulus? The active vision framework (Rolfs & Schweitzer, 2022) offers a possible explanation. The visual system is regularly exposed to translational image shifts during saccadic eye movements (Lee, Zee, & Straumann, 2000). By contrast, rotational displacements of the retinal image are minimal during natural viewing, typically limited to only 1 to 2 degrees during horizontal and vertical eye movements (Straumann, Zee, Solomon, Lasker, & Roberts, 1995). This asymmetry between the frequency of translational and rotational image motions may explain why the visual system is better equipped to handle distortions generated by translation, thereby preserving shape structure in the case of saccades and the frame stimulus. In contrast, the rotational motion in the flash-grab stimulus may not engage the same stabilizing mechanisms and instead reveals the disruptive effects of motion-induced shifts on perceived shape.

To account for the difference in shape distortions between the flash-grab and frame effects, we can consider three possibilities. First, rotational motion may produce spatially nonuniform local shifts relative to the moving background contour that lead to distortions (Adamian et al., 2023), whereas linear motion produces spatially uniform effects so that probes are displaced as a whole but not distorted. The results of Experiment 2 argue against this explanation. The predicted distortions based on remembered locations of individual elements of the probe T did not match the observed distortions measured by online adjustments. Although these failures in the predictions may be due to the different measurement techniques, they are more likely due to the involvement of more global factors underlying the distortions.

Second, these differential effects of rotational and translational motion may arise because rotational motion acts at a stage before the extraction of shape so that any differential local shifts lead to shape distortion. In contrast, the effects of linear motion may emerge later, at a level beyond the establishment of shape, where the frame's motion shifts the whole shape without distorting it. A similar case is seen for the effects of saccades on position and shape. Specifically, the perceived location of probes briefly flashed around the time of the saccade are compressed toward the saccade target (Morrone, Ross, & Burr, 1997; Ross, Morrone, & Burr, 1997). However, saccadic compression affects the position, but not the shapes of the flashed probes. When multiple squares are presented, for example, their spacing is compressed but, importantly, the width of each square itself is unchanged (Matsumiya & Uchikawa, 2001). This absence of shape distortion in the presence of large position shifts suggests that the perisaccadic position shifts act on individual objects at a level beyond the establishment of shape. The same may be the case for the frame effect.

Third, it is possible that, regardless of the type of motion, the shape representation is spatially distorted at early stages of visual processing. This result is consistent with our finding that predicted shape distortion (based on localized probe dots) was similar across stimuli (Figure 6). However, in the case of translational motion, the original shape may be recovered at later stages either by reversing the local distortions or by transforming it to the closest familiar shape (Sinha & Poggio, 1996). This shape recovery process would have evolved to preserve stimulus shapes across frequent saccades, where any significant shape distortion would disrupt visual stability with each eye movement.

Although we cannot yet distinguish among these alternatives, the current findings suggest that the frame stimulus, perhaps owing to its resemblance to the effects of natural saccadic motion, benefits from a compensatory mechanism, resulting in greater shape preservation. The flash-grab stimulus, with its rotational motion within a circular aperture, does not, producing stronger shape distortions. This distinction reveals a potential dissociation between local position coding and global shape perception and suggests that perceptual stabilization mechanisms may be selectively engaged when motion trajectories resemble the lawful kinematics of self-generated visual motion.

## Supplementary Material

Supplement 1

Supplement 2

Supplement 3

Supplement 4

Supplement 5

Supplement 6

Supplement 7

Supplement 8

Supplement 9

## Supplementary material

Supplementary Movie S1. The flash-grab effect. The red probe flashes at the vertical location but may appear shifted leftward in the direction of the motion following the flash (Cavanagh & Anstis, 2013). Please use this link to download the movie: https://osf.io/8uywg/files/95x6e.

Supplementary Movie S2. The frame effect. The red and blue probes are flashed at the same location on the screen, but the blue probe may appear shifted to the right of the red probe (Özkan et al., 2021). The blue probe is at the right edge of the frame when it flashes, and the red probe is at the left of the frame when it flashes. The perceived separation between the probes suggests that they are perceived in the frame's coordinate space. Please use this link to download the movie: https://osf.io/8uywg/files/t4be9.

Supplementary Movie S3. The frame condition in Experiment 1. Fixate the black spot and judge the location of the horizontal component of the flashed T relative to its vertical component. Participants adjusted the position of the horizontal component of the reference T on the right to match their perception of the flashed T on the left. If afterimages are noticeable, the effects in the movies may be severely reduced. Please use this link to download the movie: https://osf.io/8uywg/files/m2hdk.

Supplementary Movie S4. The flash-grab condition in Experiment 1. Fixate the black spot and judge the location of the horizontal component of the flashed T relative to its vertical component. Participants adjusted the position of the horizontal component of the reference T on the right to match their perception of the flashed T on the left. If afterimages are noticeable, the effects in the movies may be severely reduced. Please use this link to download the movie: https://osf.io/8uywg/files/u6g3d.

Supplementary Movie S5. The frame condition in Experiment 2. In this sample trial the probe flashes at the middle location of the virtual T. Once the display was erased, participants used a mouse to click on the remembered location of the probe. Please use this link to download the movie: https://osf.io/8uywg/files/qakmx.

Supplementary Movie S6. The frame condition in Experiment 2. In this sample trial the probe flashes at the middle location of the virtual T. Once the display was erased, participants used a mouse to click on the remembered location of the probe. Please use this link to download the movie: https://osf.io/8uywg/files/9nt5k.

Supplementary Movie S7. Rotating cross inside a rectangular aperture in Experiment 3. Fixate the black spot and judge the location of the horizontal component of the flashed T relative to its vertical component. Participants adjusted the position of the horizontal component of the reference T on the right to match their perception of the flashed T on the left. If afterimages are noticeable, the effects in the movies may be severely reduced. Please use this link to download the movie: https://osf.io/8uywg/files/yhcp9.

Supplementary Movie S8. Translating square inside a circular aperture in Experiment 3. Fixate the black spot and judge the location of the horizontal component of the flashed T relative to its vertical component. Participants adjusted the position of the horizontal component of the reference T on the right to match their perception of the flashed T on the left. If afterimages are noticeable, the effects in the movies may be severely reduced. Please use this link to download the movie: https://osf.io/8uywg/files/yedg5.

Supplementary Movie S9. Translating square inside a rectangular aperture in Experiment 3. Fixate the black spot and judge the location of the horizontal component of the flashed T relative to its vertical component. Participants adjusted the position of the horizontal component of the reference T on the right to match their perception of the flashed T on the left. If afterimages are noticeable, the effects in the movies may be severely reduced. Please use this link to download the movie: https://osf.io/8uywg/files/dcr5y.
